# Arterial Perivascular Space‐Mediated Solute Transport in the Mouse Brain

**DOI:** 10.1002/EXP.20250264

**Published:** 2026-06-19

**Authors:** Shiyong Li, Ye Wang, Guangyu Jia, Yamei Yu, Zhe Zhang, Qi Wang, Kaelyn V. Becker, Jonathan W. Engle, Dawei Jiang, Weibo Cai

**Affiliations:** ^1^ Department of Neurology Second Affiliated Hospital of Nanchang University Jiangxi Medical College Nanchang University Nanchang China; ^2^ Jiangxi Province Key Laboratory of Neurological Diseases Nanchang China; ^3^ Departments of Radiology and Medical Physics University of Wisconsin ‐ Madison Madison Wisconsin USA; ^4^ Department of Nuclear Medicine Union Hospital Tongji Medical College Huazhong University of Science and Technology Wuhan China

**Keywords:** cerebrospinal fluid (CSF), interstitial fluid (ISF), perivascular space (PVS)

## Abstract

The perivascular space (PVS) around cerebral arteries supports nutrient delivery and waste clearance, yet its transport mechanisms remain poorly defined. Here, we establish arterial PVS as a major solute transport route in the brain, exhibiting marked region‐specific functionality. Using in vivo imaging, we show that solute movement within arterial PVS is not driven by cerebrospinal fluid (CSF) bulk flow, but follows bidirectional, arterial‐pulsation‐dependent dynamics. Parenchymal solutes diffuse into the vascular PVS network, where arterial pulsations propel them along perivascular pathways toward capillary and venous compartments to facilitate clearance. Notably, after cortical injection, reduced arterial pulsation and blood flow following carotid ligation significantly increased Aβ retention within arterial PVS. These results define a pulsation‐driven perivascular transport system essential for brain solute clearance in health and disease.

## Graphical abstract

The mechanisms underlying solute clearance from the brain parenchyma remain debated, with competing hypotheses involving bulk cerebrospinal fluid flow versus perivascular transport. Using multimodal in vivo imaging and computational modeling in mice, this study demonstrates that arterial pulsation drives bidirectional solute movement within the perivascular space, facilitating net transport of molecules such as amyloid‐beta toward capillary and venous compartments independently of bulk CSF flow. These findings establish arterial‐pulsation‐dependent perivascular transport as a key clearance pathway in the brain, with impaired pulsation leading to solute retention, which has direct implications for understanding waste accumulation in neurodegenerative diseases.

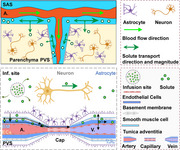

## 1 Introduction

Efficient transport of solutes in cerebrospinal fluid (CSF) and interstitial fluid (ISF) is vital for maintaining brain homeostasis, supporting highenergy needs, and facilitating rapid elimination of metabolic wastes [1, 2, 3, 4, 5, 6, 7, 8, 9, 10]. Traditional theories suggest that CSF primarily drives solute transfer; however, recent findings have challenged this perspective [11, 12, 13, 14, 15, 16, 17]. The glymphatic hypothesis proposes that CSF enters the brain along cerebral arteries, mixes with ISF, and exits through veins, highlighting the CSF‐ISF exchange process [18, 19, 20]. Additionally, recent discoveries underscore the significant role of meningeal lymphatic vessels in facilitating solute drainage within CSF and ISF [8, 21, 22], together with extracranial pathways such as the nasopharyngeal lymphatic plexus and deep cervical lymphatic vessels [23]. Despite this, the validity of the glymphatic hypothesis remains a topic of debate. For example, Smith et al. demonstrated that genetic deletion of aquaporin‐4 (AQP4) does not impair solute entry from the subarachnoid space (SAS) into the brain parenchyma [24], thereby challenging the central role of astrocytic water channels in CSF‐ISF exchange. Moreover, the mechanisms by which metabolic waste is transported to the SAS in brain regions lacking lymphatic vessels remain poorly understood [24, 25, 26].

Furthermore, the perivascular space (PVS), also known as the Virchow‐Robin space, is recognized as vital for CSF and ISF exchange and for the clearance of metabolic waste. Under pathological conditions such as ischemia, CSF may aberrantly enter the brain parenchyma via PVS, thereby contributing to cerebral edema formation [27]. However, the specific mechanisms regulating solute transport within the arterial PVS are not well understood [28, 29], and it remains uncertain whether these processes vary across different brain regions [1, 5, 9, 30, 31, 32].

To address these challenges, we employed a multifaceted approach that accounts for regional variations across the brain. By utilizing multiple tracers, diverse tracer infusion sites, and advanced imaging techniques, including positron emission tomography/computed tomography (PET‐CT), live‐cell tracking, and 3DISCO imaging with enhanced fluorescence preservation, we aimed to elucidate the mechanisms of solute transport within the arterial PVS. Additionally, we seek to clarify the underlying modes of action by tracing beta‐amyloid in both healthy and diseased animal models.

## 2 Results and Discussion

### 2.1 Solute Transport via Arterial PVS Varies by Brain Regions

To investigate the mechanisms of solute transport, we used a highly sensitive probe, [^64^Cu]CuCl_2_, with a half‐life of 12.7 h for non‐invasive dynamic molecular‐level imaging. Dynamic PET imaging following intravenous injection of [^64^Cu]CuCl_2_ in healthy mice revealed low radioactivity in both the bloodstream and brain, with significantly elevated levels detected in the liver and spleen (Supplementary Figures S1A–F, Movie S1). This indicates rapid blood clearance of [^64^Cu]CuCl_2_, leading to lower imaging background, and limited penetration through the blood‐brain barrier into the brain parenchyma, enabling better tracking of solute transport.

Subsequently, after intraplantar injection of [^64^Cu]CuCl_2_ (50 µCi, ∼5 µL) into the right hind paw, we observed sequential migration of [^64^Cu]CuCl_2_ through the popliteal, inguinal, and renal lymph nodes within the first 10 min post‐injection (p.i.) (Supplementary Figure S1G, H). The signal in the lymph nodes peaked at 5–7 min p.i., then gradually decreased before stabilizing (Supplementary Figure S1I‐K). The low signal of [^64^Cu]CuCl_2_ in the circulatory system, combined with its stable presence in tissues and the lymphatic system, suggests its potential as an excellent probe for elucidating clearance mechanisms of solute molecules.

In this study, to ensure the accuracy of intracranial solute transport observations, we systematically evaluated the potential off‐target effects of the tracers used on vascular reactivity and PVS dynamics. The injection dose of [^64^Cu]CuCl_2_ used in the experiments was 2 µL (1‐2 µCi, approximately 1.6 nM), which is significantly lower than the physiological concentration of copper ions in CSF [33] and far below the pro‐inflammatory threshold required to induce acute endothelial barrier disruption or inflammatory responses. Therefore, its impact on vascular permeability, vascular tone, and PVS dynamics is negligible. Additionally, blood and urine samples, along with histopathological examination of the heart, liver, spleen, lungs, and kidneys following intravenous injection of [^64^Cu]CuCl_2_ in healthy mice, demonstrated its good biocompatibility (Supplementary Figure S2). Building on these findings, we employed [^64^Cu]CuCl_2_ to explore solute transport routes within the parenchyma, ventricular system (intraventricular), and SAS (extraventricular).

**FIGURE 1:**
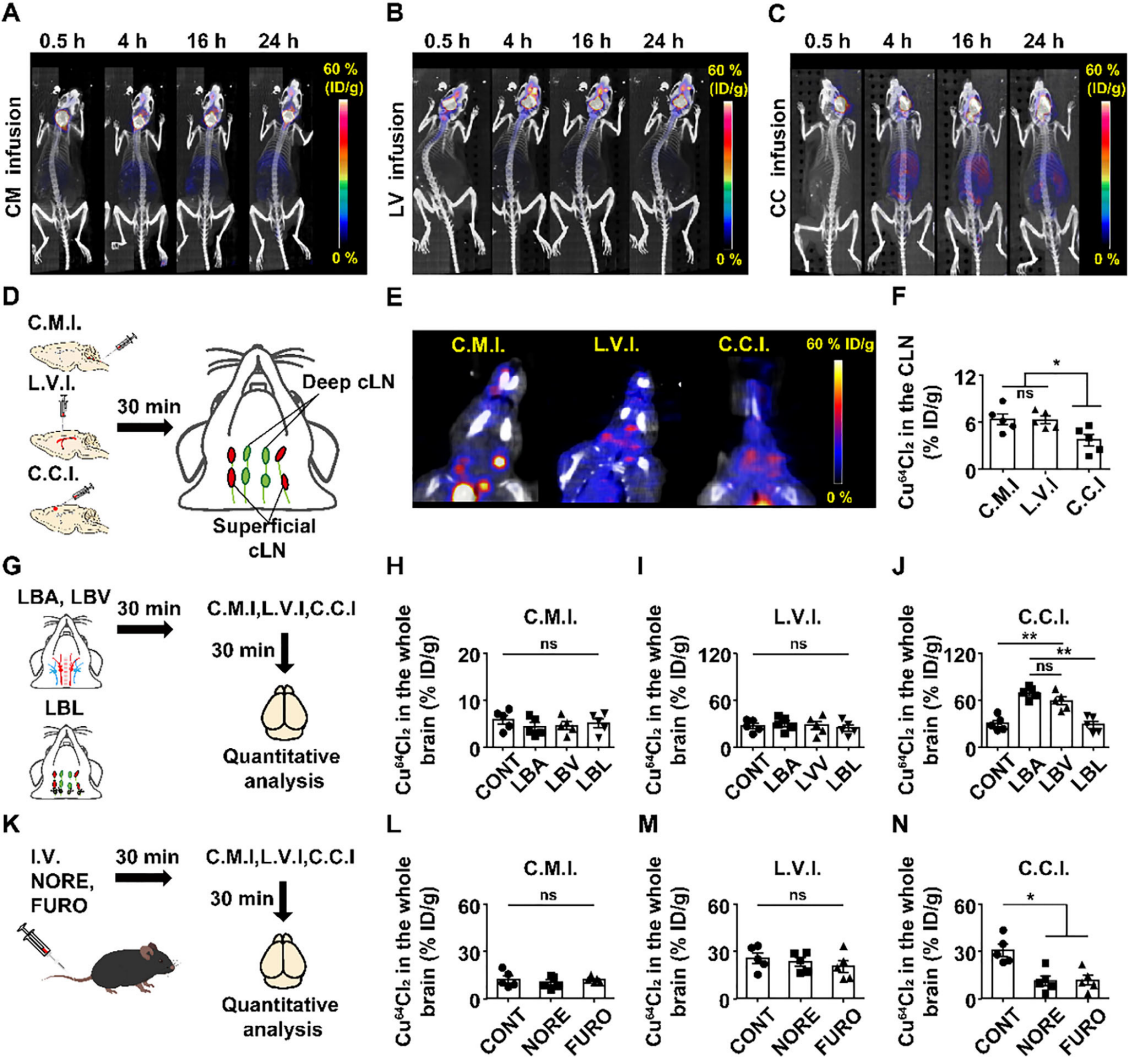
The [^64^Cu]CuCl_2_ drainage and clearance across varying brain regions. Representative whole body PET/CT images at 0.5, 4, 16, and 24 h after cisterna magna (A), lateral ventricle (B), and cerebral cortical (C) infusion. (D) Schematic and (E) PET‐CT imaging of [^64^Cu]CuCl_2_ in cervical lymph nodes (including superficial and deep cervical LNs) at 30 min post‐infusion. (F) Quantitative analysis of [^64^Cu]CuCl_2_ signal in the cervical lymph nodes (n = 5, mean ± SD, *p < 0.05, Student's t‐test). (G) Schematic showing infusions into varying brain regions at 30 min post‐ligation, followed by whole‐brain quantitative analysis. For cisterna magna infusion (H) and lateral ventricle infusion (I), LBA, LBV, and LBL did not increase [^64^Cu]CuCl_2_ retention in the brain (n = 5). (J) For cerebral cortical infusion, LBA and LBV did not increase [^64^Cu]CuCl_2_ retention in the brain (n = 5, mean ± SD, *p < 0.05, **p < 0.01, Student's t‐test). (K) Schematic illustrating infusions into various brain regions at 30 min post‐ligation, followed by whole‐brain quantitative analysis. For cisterna magna infusion (L) and lateral ventricle infusion (M), norepinephrine and furosemide did not increase [^64^Cu]CuCl_2_ retention in the brain (n = 5, mean ± SD, *p < 0.05, Student's t‐test). (n) For cerebral cortical infusion, norepinephrine and furosemide significantly increased [^64^Cu]CuCl_2_ retention in the brain (n = 5, mean ± SD, *p < 0.05, Student's t‐test). Abbreviations: C.M.I., cisterna magna infusion; L.V.I.: lateral ventricle infusion; C.C.I., cerebral cortical infusion; LNs: lymph nodes; LBL: ligation of bilateral cervical lymphatic vessels; LBV: ligation of bilateral carotid jugular veins; LBA: ligation of bilateral carotid arteries; *Note*: norepinephrine; Furo: furosemide.

A cisterna magna infusion was performed to investigate solute transport pathways in the SAS. Within 0–24 h post infusion, PET‐CT maximum intensity projection (MIP) imaging revealed the dispersion of [^64^Cu]CuCl_2_ from the cisterna magna towards the cranial base and spinal cord (Figure 1A). At 30 min post cisterna magna infusion, PET‐CT imaging and quantitative analysis indicated that less than 5% of [^64^Cu]CuCl_2_ signal was detected in the cortex and deep brain nuclei and approximately 10% in the olfactory bulbs, while ∼70% was cleared extracranially (Supplementary Figure S3A–D). These findings suggest that less than 5% of [^64^Cu]CuCl_2_ penetrates the brain parenchyma, while over 80% rapidly exits via the meningeal lymphatic system, the olfactory bulbs, and the spinal cord.

To further investigate the pathway of metal ion transport within the lateral ventricle, we performed a ^64^Cu infusion, and at 24 h after which, the radioactive signal rapidly distributes from the injection site through the entire ventricular system (Figure 1B). Within the initial 30 min following infusion, [^64^Cu]CuCl_2_ was detected in bilateral deep brain tissue, the intra‐ventricular system, and the cisterna magna, indicating the metal ions' ability to penetrate the ventricular walls and transport through the central ventricular aqueduct to the SAS (Supplementary Figure S3E–H). Moreover, dynamic PET imaging and quantitative analysis revealed a rapid transit of [^64^Cu]CuCl_2_ to the SAS via the ventricular system, with over 70% exiting the brain. These findings suggest an efficient solute transport mechanism from the intraventricular system to the SAS (Supplementary Figure S3I,J).

The pathways of solute transport in the brain parenchyma were investigated through cerebral cortical infusion. Within 0–24 h post‐infusion, PET‐CT images demonstrated the initial distribution of [^64^Cu]CuCl_2_ in the ipsilateral cortical parenchyma and the corresponding SAS, followed by a broader dispersion throughout the SAS (Figure 1C). This highlights the SAS as a crucial pathway for solute transport from the cortical parenchyma. By 30 min post cerebral cortical infusion, approximately 1% of [^64^Cu]CuCl_2_ remained in the cortex, 5% in the olfactory bulbs, and less than 3% in the deep brain nuclei and intraventricular system (Supplementary Figure S3K–N), suggesting a possible pathway connecting the cortex to the lateral ventricles and deep brain nuclei.

Cervical lymph nodes are known to communicate with meningeal lymphatic vessels, playing a crucial role in clearing metabolic waste from the brain [2, 34, 35, 36]. We hypothesized that the role of the meningeal lymphatic system in draining solute molecules from various brain regions could be elucidated by measuring changes in [^64^Cu]CuCl_2_ levels in cervical lymph nodes (Figure 1D). At 30 min after infusion, cervical lymph node [^6^ ^4^Cu]CuCl_2_ levels were significantly higher after cisterna magna or intracerebroventricular injection than after cortical infusion, indicating faster solute drainage from the subarachnoid and ventricular spaces than from the brain parenchyma (Figure 1E,F).

To explore the contributions of lymphatic vessels, the cerebral arterial system, and the venous system in regulating brain solute clearance, we separately ligated bilateral cervical lymphatic vessels (BCLV), bilateral carotid jugular veins (BCJV), and bilateral carotid arteries (BCA). Following these ligations, we conducted tracer infusion at different sites and quantitatively analyzed the remaining [^64^Cu]CuCl_2_ in the brain (Figure 1G). Our findings demonstrate that following infusion from the cisterna magna and lateral ventricles, ligation of BCLV, BCJV, and BCA did not increase the residual [^64^Cu]CuCl_2_ levels in the brain (Figure 1H,I). This suggests that solute clearance from both the intraventricular and extraventricle involves multiple pathways. In contrast, after cerebral cortical infusion, ligation of the BCJV and BCA significantly enhanced [^64^Cu]CuCl_2_ retention in the brain, while ligation of BCLV showed no effect (Figure 1J). These results indicate that the arterial system plays a critical role, alongside the venous system, in clearing solutes from the brain parenchyma.

Norepinephrine enhances arterial pulsatility via α_1_/β_1_‐adrenergic activation, whereas furosemide induces prostaglandin‐mediated venodilation that reduces cardiac preload and alters venous return [37, 38, 39, 40]. To confirm that regulating the arterial and venous systems alone can clear [^64^Cu]CuCl_2_ from the brain parenchyma rather than the ventricular system and SAS, we administered norepinephrine to enhance arterial pulsation and furosemide to promote venous reflux (Figure 1K). Following cisterna magna and lateral ventricle infusion, neither norepinephrine nor furosemide reduced the residual [^64^Cu]CuCl_2_ levels in the brain. In contrast, after cerebral cortical infusion, both norepinephrine and furosemide effectively decreased the residual [^64^Cu]CuCl_2_ in the brain (Figure 1L–N). Notably, after cortex infusion, either norepinephrine or furosemide treatment alone significantly reduced [^64^Cu]CuCl_2_ retention in the brain parenchyma.

These data suggest that in the brain parenchyma, the circulatory system is the primary mechanism for clearing [^64^Cu]CuCl_2_. In contrast, both the circulatory and lymphatic systems contribute to solute clearance in the CSF, including the SAS and ventricular system. Despite extensive research highlighting the crucial role of PVS in clearing metabolic waste from the brain parenchyma, the underlying mechanisms remain unclear. Therefore, we aim to further investigate the role and mechanisms of arterial PVS in solute transport within the brain parenchyma.

### 2.2 The Role of Arterial PVS in Clearing Solutes From the Brain Parenchyma

**FIGURE 2:**
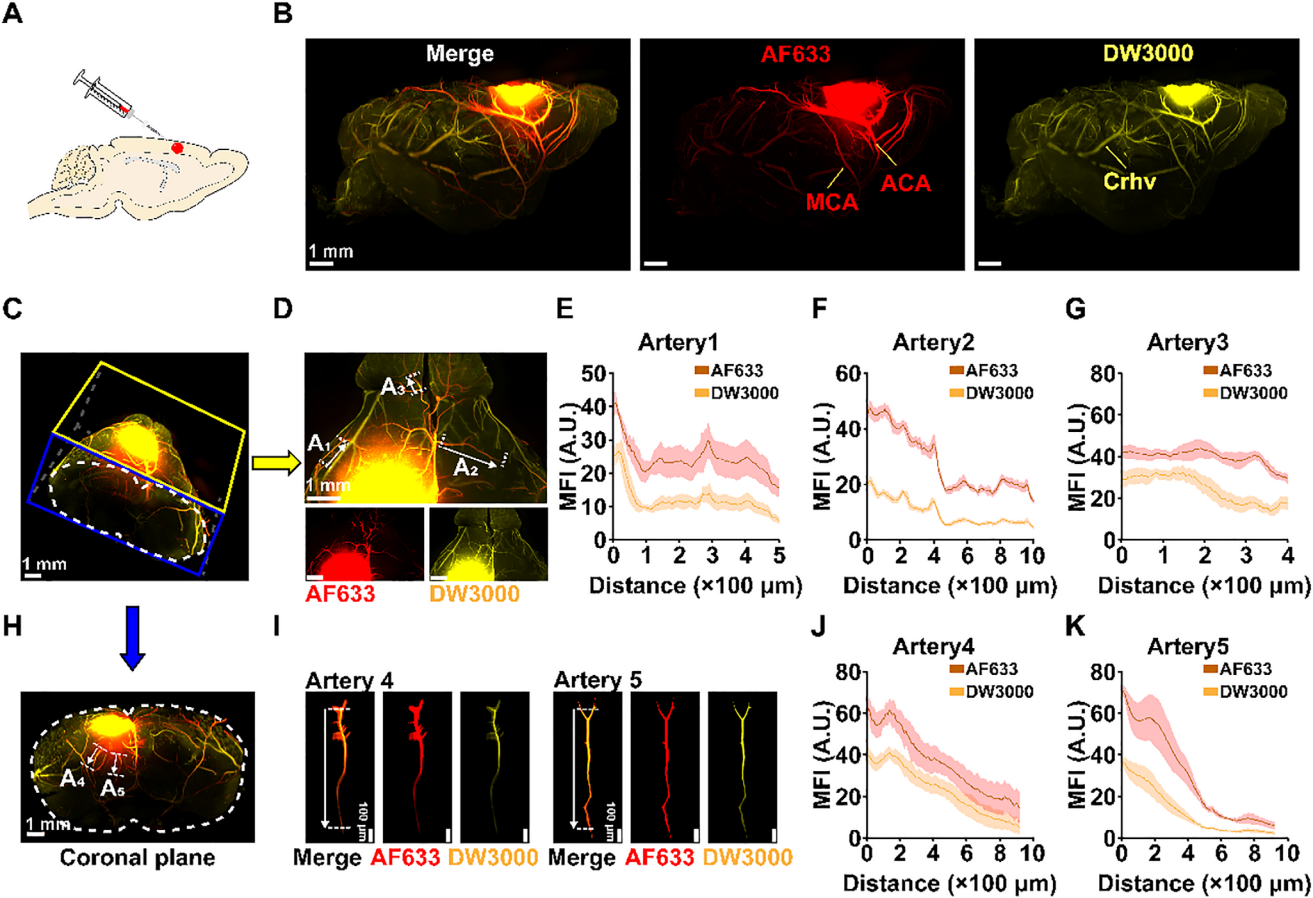
The arterial PVS is a critical pathway for clearing solutes from the cortical parenchyma. (A) Schematic diagram of 3DISCO images was acquired at 30 min after cerebral cortical infusion with the mixture of fluorescent molecules (AF633, red; DW3000, yellow). (B) The distribution of DW3000 and AF633 in the whole brain. (C) Observation from the rear and magnified examination of sliced 3D brain tissue. (D) The accumulation of DW3000 and AF633 in arteries 1, 2, and 3 (A1, A2, and A3). Quantification of DW3000 and AF633 mean fluorescence intensity (MFI) aligned to the onset of the SD for artery 1 (E), artery 2 (F), and artery 3 (G). (H) Observation of dye molecule distribution in 3D brain tissue slices from coronal sections. (I) The dye molecule distribution within the brain parenchyma, in Artery 1 (arrow 1) and Artery 2 (arrow 2) in Figure 2 H. Distance series of MFI in Arteries 4 (J), 5 (K). Abbreviation: ACA, anterior cerebral artery; AF633, Alexa Fluor 633 hydrazide; Crhv, Caudal rhinal vein; CSF, Cerebrospinal fluid; DW3000, Dextran Texas Red 3000; ISF, interstitial fluid; MCA, middle cerebral arteries; MFI, mean fluorescence intensity; PVS, perivascular space; Rrhv, Rostral rhinal vein.

While the arterial PVS forms a critical pathway for CSF influx from the SAS into the brain parenchyma [41, 42], its role in the drainage of metabolic waste remains debated. To reveal the role of arterial PVS in solute clearance, we infused a mixture of AF633 and DW3000 dyes (1 µL, 10 min infusion) into the cortical parenchyma to monitor arteries and neurons, respectively [43, 44]. Following cortical injection, 3DISCO imaging after 30 min revealed that AF633‐labeled arteries were present within the brain parenchyma and on its surface, including the arterial PVS of the anterior cerebral artery (ACA), the middle cerebral arteries (MCAs), and their branches, extending beyond the injection site (Figure 2A,B). Additionally, DW3000 molecules were detected at the injection site and within the arterial PVS (MCA and its branches) and the venous PVS (rostral rhinal vein, caudal rhinal vein, and their branches). These findings indicate the involvement of both arterial and venous PVS in the clearance of dye molecules within the brain parenchyma.

To assess the clearance pattern of dye molecules in arterial PVS on the brain surface, we analyzed the intensity fluctuations of AF633 and DW3000 along three dorsal arterial branches (Figure 2C,D). Fluorescence intensity of AF633 and DW3000 gradually decreases along Artery 1 (a branch of the MCA) and Artery 2 (the medial branch of the anterior cerebral artery). Artery 3 (a branch of the olfactory artery) as the distance from the injection site increased (following the direction of the arrows), suggesting that dye molecules can propagate along the arterial PVS in the direction of blood flow on the brain surface (Figure 2E–G).

Furthermore, we assessed changes in fluorescence intensity of AF633 and DW3000 in cortical parenchymal arterial vessels. Both AF633 and DW3000 exhibited high‐intensity signals surrounding the injection site, indicating that the initially infused dye molecules create a localized high‐concentration area before spreading to adjacent brain regions (Figure 2H). In Artery 4 and 5 (with AF633‐positive staining), the fluorescence intensity of both dyes gradually decreased with increasing distance from the injection site (Figure 2I–K). These data demonstrate that dye molecules disperse within the deep brain parenchyma, enter the arterial PVS, and propagate against the direction of blood flow.

Further, we observed a significant reduction in the distribution and fluorescence intensity of AF633 and DW3000 in the brain parenchyma at 90 min post‐injection (Supplementary Figure S4A). Compared to 30 min, both parameters markedly decreased, indicating adequate, time‐dependent clearance (Supplementary Figure S4B, C). Signals of AF633 and DW3000 near the ACA at the injection site were strong, but absent in the Willis circle, suggesting limited propagation beyond the immediate vicinity. Distribution analysis revealed that at 90 min, AF633 levels remained stable due to its high arterial affinity, whereas DW3000 levels declined significantly, indicating rapid extravasation and clearance from the arterial periphery (Supplementary Figure S4D–K).

Consistent with previous findings, small‐molecule transport within the brain parenchyma occurs by diffusion. Horizontal sections showed elliptical distributions of AF633 and DW3000 that extended into adjacent PVS and cleared in a time‐dependent manner (Supplementary Figure S5A,B), indicating diffusion from the injection site and subsequent removal. Confocal microscopy revealed colocalization of DW3000 (yellow) with the PVS of arteries (red) and the basement membrane (green) of capillaries and small vessels (Supplementary Figure S5C), demonstrating that solutes can diffuse into PVS and penetrate the endothelial basement membrane of microvasculature (Supplementary Figure S5D).

Aquaporin‐4 (AQP4) facilitates water movement across cell membranes in response to osmotic gradients [45], but its effect on solute diffusion in the brain extracellular space (ECS)‐important for brain function and drug delivery‐remains uncertain. Here, we used dynamic PET imaging to compare the diffusion of ^6^ ^4^Cu^2^^+^ in mice treated with an AQP4 inhibitor and in controls. Both groups showed nearly identical, radially symmetric diffusion from the injection site, with no significant differences in activity–distance profiles (*p* > 0.05) in either coronal or horizontal planes. These findings indicate that while AQP4 regulates water transport, it does not affect solute diffusion in the brain ECS (Supplementary Figure S6A–F).

### 2.3 Simulated Model of Solute Influx and Efflux in the Arterial PVS

**FIGURE 3:**
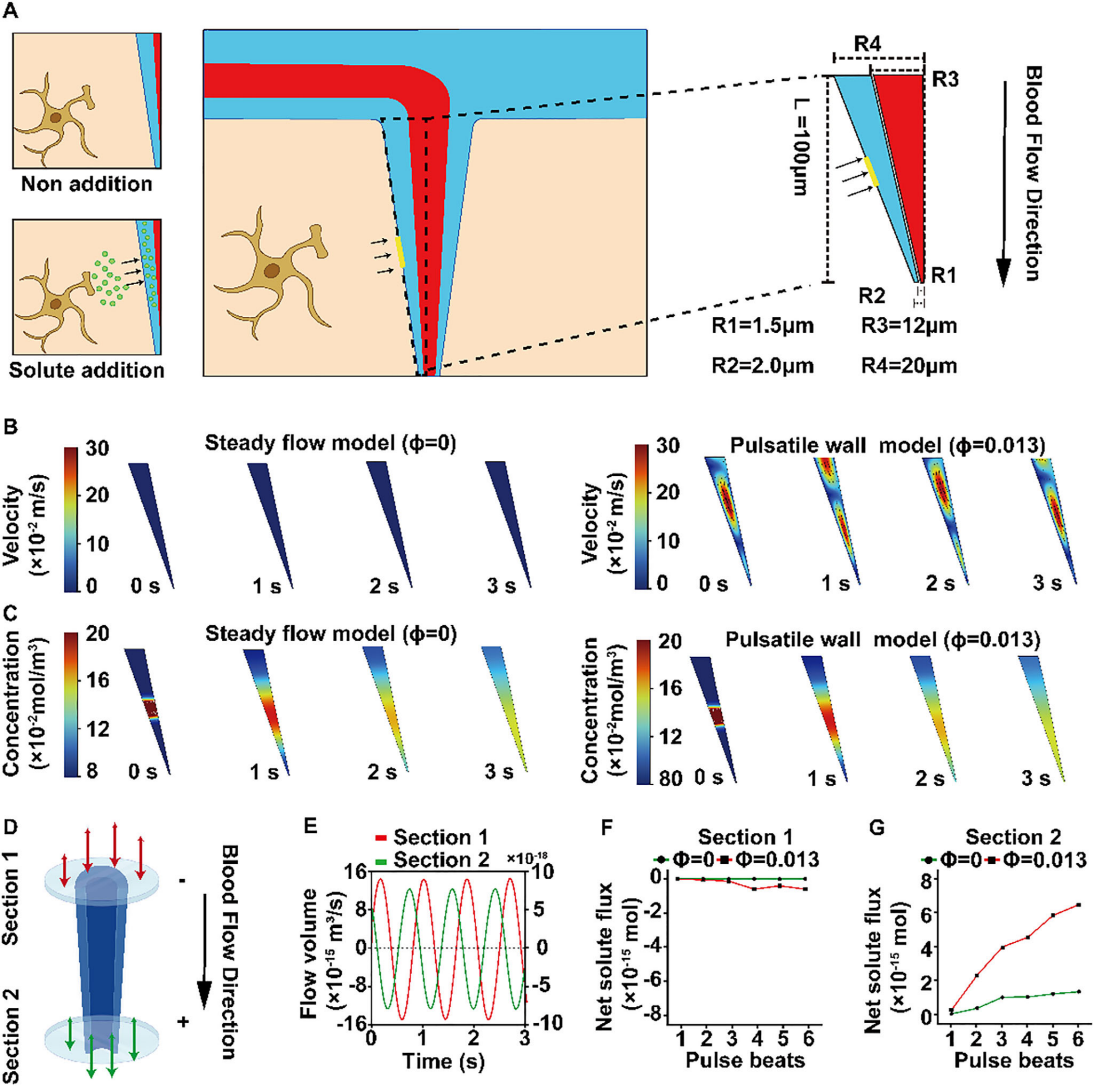
Simulations of solute movement within arterial perivascular space (PVS) in the cortical parenchyma. (A) Arterial PVS model geometry constructed based on physiological parameters of the cerebrovascular system. (B) Two‐dimensional simulations of cerebrospinal fluid (CSF) flow within the PVS under non‐pulsatile (*φ* = 0) and arterial pulsation (*φ* = 0.013) conditions. (C) Solute distribution following introduction at the PVS midpoint (left) and subsequent solute movement (right) under non‐pulsatile (*φ* = 0) and pulsatile *(φ* = 0.013) conditions. (D) Schematic defining Cross‐Sections 1 and 2 at the ends of the arterial perivascular space (PVS), with the sign convention for flow direction (positive downstream, negative upstream). (E) Instantaneous cerebrospinal fluid (CSF) flux across Section 1 (red) and Section 2 (green) over 0–3 s. (F) Cumulative net solute flux across Section 1. (G) Cumulative net solute flux across Section 2.

We developed a computational model to elucidate solute transport mechanisms within arterial PVS. An arterial PVS geometry was constructed based on physiological parameters of the cerebrovascular system [46], simulating pulsation‐driven CSF flow (Figure 3A). The wider PVS end was defined as the afferent (inflow) end and the narrower end as the efferent (outflow) end, jointly defining the principal axis for CSF and solute transport. Under both non‐pulsatile (*φ* = 0) and arterial pulsation (*φ* = 0.013) conditions, the two‐dimensional CSF flow field within the PVS exhibited bidirectional oscillations synchronized with the cardiac cycle (Figure 3B). Solute was introduced at the PVS midpoint; its distribution under both conditions is shown schematically (Figure 3C). In this model, Cross‐Sections 1 and 2 were defined to establish the principal axis for solute transport. The flow direction was aligned with blood flow (positive downstream, negative upstream; Figure 3D). Analysis of the instantaneous CSF flux over 0–3 s revealed bidirectional oscillatory flow at both sections, synchronized with the cardiac cycle and exhibiting differences in amplitude and phase (Figure 3E). Calculation of the net solute flux, however, demonstrated a net efflux at both sections, with the flux at Section 2 being significantly greater than that at Section 1 (Figure 3F,G). These results indicate that arterial pulsations enhance the bidirectional transport of solutes within the arterial PVS and promote a net movement of solutes from the arterial PVS towards the capillary basement membrane. We propose a solute transport model jointly driven by arterial pulsation and basement‐membrane concentration gradients. In this model, solute movement within the arterial PVS is distinct from bulk CSF flow. Arterial pulsation enhances bidirectional mixing, drives solutes from the arterial PVS toward the capillaries, and facilitates their entry into the capillary basement membranes, where exchange with the blood and ISF occurs. (Supplementary Figure S6G).

### 2.4 Arterial PVS Serves as the Entry Into the Brain Parenchyma

**FIGURE 4:**
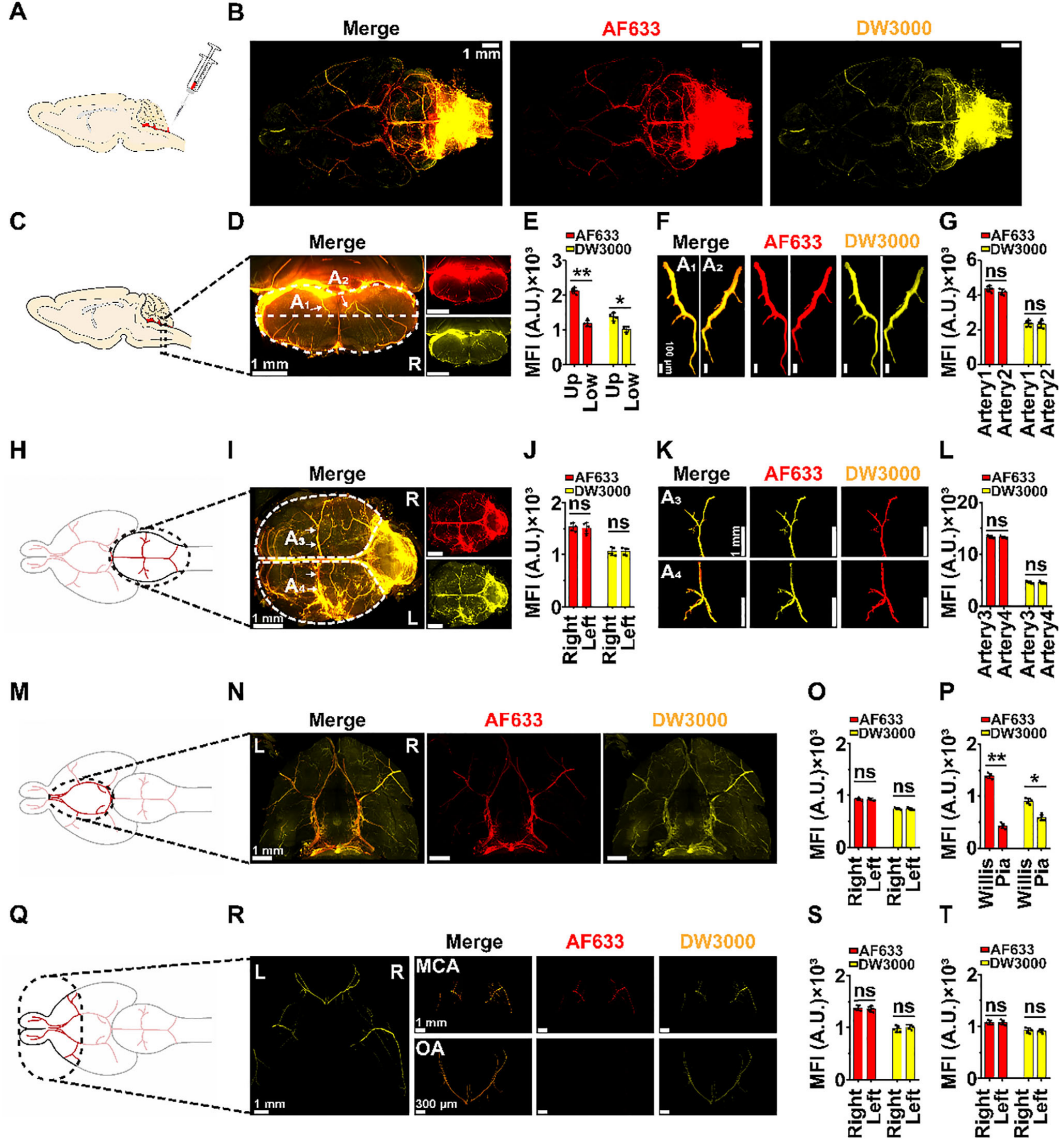
The arterial PVS is a critical pathway for solute entry into the central nervous system parenchyma. (A) Schematic diagram of 3DISCO images was acquired at 30 min after C.M.I. with the mixture of fluorescent molecules (AF633, red; DW3000, yellow). (B) The distribution of DW3000 and AF633 in the whole brain. (C) Schematic diagram of 3DISCO images of the analyzed 3D spinal cord sections. (D) 3DISCO images of 3D spinal cord sections. (E) Quantitative analysis of MFI in dorsal (above dashed line) and ventral regions of 3D spinal cord sections (n = 5, mean ± SD, *p < 0.05, Student's t‐test). (F) Magnified observation of images of Artery 1 and Artery 2. (G) Quantitative analysis of MFI in Artery 1 (left of spinal cord midline) and Artery 2 (right of spinal cord midline). (H) Schematic of 3DISCO images of the brainstem's ventral aspect. (I) Distribution of dye molecules in the ventral aspect of the brainstem (J) and quantitative analysis. (K) Higher‐magnification images and (L) MFI of Arteries 3 and 4. (M) Ventral view diagram of the Circle of Willis. (N) Distribution of dye molecules in the Circle of Willis. (O) Overall fluorescence intensity is symmetrical between hemispheres. (P) Fluorescence intensity in arterial and meningeal regions at the circle of Willis level (n = 5, mean ± SD; *p < 0.05, **p < 0.01, Student's t‐test). (Q) Schematic diagram of MCA and OA. (R) Distribution of dye molecules in the MCA and OA. (S) Fluorescence intensity in the left vs. right middle cerebral artery (MCA). (T) Fluorescence intensity in left vs. right olfactory artery (OA). Abbreviation: AcomA, anterior communicating artery; BA, basilar artery; MFI, mean fluorescence intensity; OA, Olfactory Bulb Artery; PcomA, posterior communicating artery; PCA, posterior cerebral artery.

Prior research has highlighted the crucial role of arterial PVS in facilitating the entry of solute molecules from the SAS into the brain parenchyma, but the mechanisms remain unclear [30, 47]. Here, 3DISCO imaging showed the signals of AF633 and DW3000 in the dorsal cerebellum, ventral brainstem, skull base, and spinal cord region post cisterna magna infusion (Figure 4A,B). In transverse spinal cord sections, AF633 and DW3000 signals were abundant in the parenchyma, blood vessels, and surface, suggesting their penetration through the pia and entry into the spinal cord parenchyma via arterial PVS (Figure 4C,D). Due to the injection site being located on the dorsal aspect of the spinal cord, the mean fluorescence intensity (MFI) of AF633 and DW3000 was higher in the dorsal spinal cord compared to the ventral spinal cord (Figure 4E). In addition, high signal intensities of AF633 and DW3000 appeared in arteries 1 (left) and 2 (right) of the dorsal spinal cord with no significant difference (Figure 4F,G), indicating that dye molecules can enter the parenchyma from the SAS via arterial PVS.

Furthermore, we investigate the role of arterial PVS in solute transport in the ventral brainstem (VB). Our findings showed that AF633 and DW3000 signals were present in the vertebral pia and arteries (Figure 4H). The arterial PVS showed significantly higher fluorescence intensity of AF633 and DW3000 compared to the pia (Figure 4I,J), with no difference between the left and right brainstem arteries (Figure 4K,L), suggesting that the arterial PVS is a crucial pathway for solute transport in the ventral brainstem. Notably, AF633 and DW3000 signals were also detected in the circle of Willis, with significantly higher intensity compared to the pia. Consistently, no significant difference in fluorescence intensity between both sides of the circle of Willis (Figure 4M‐P). Additionally, AF633 and DW3000 signals were observed in the MCAs and olfactory artery bilaterally, with no significant difference in average fluorescence intensity between the MCAs and olfactory artery on either side of the brain (Figure 4Q‐S). Consistent with previous studies [48], AF633 and DW3000 accumulated in the PVS surrounding arterial vessels and, after enrichment in this compartment, propagated along the arterial wall, ultimately translocating toward and reaching the nasal mucosa.

### 2.5 Modeled Solute Exchange at the SAS‐arterial PVS Interface

**FIGURE 5:**
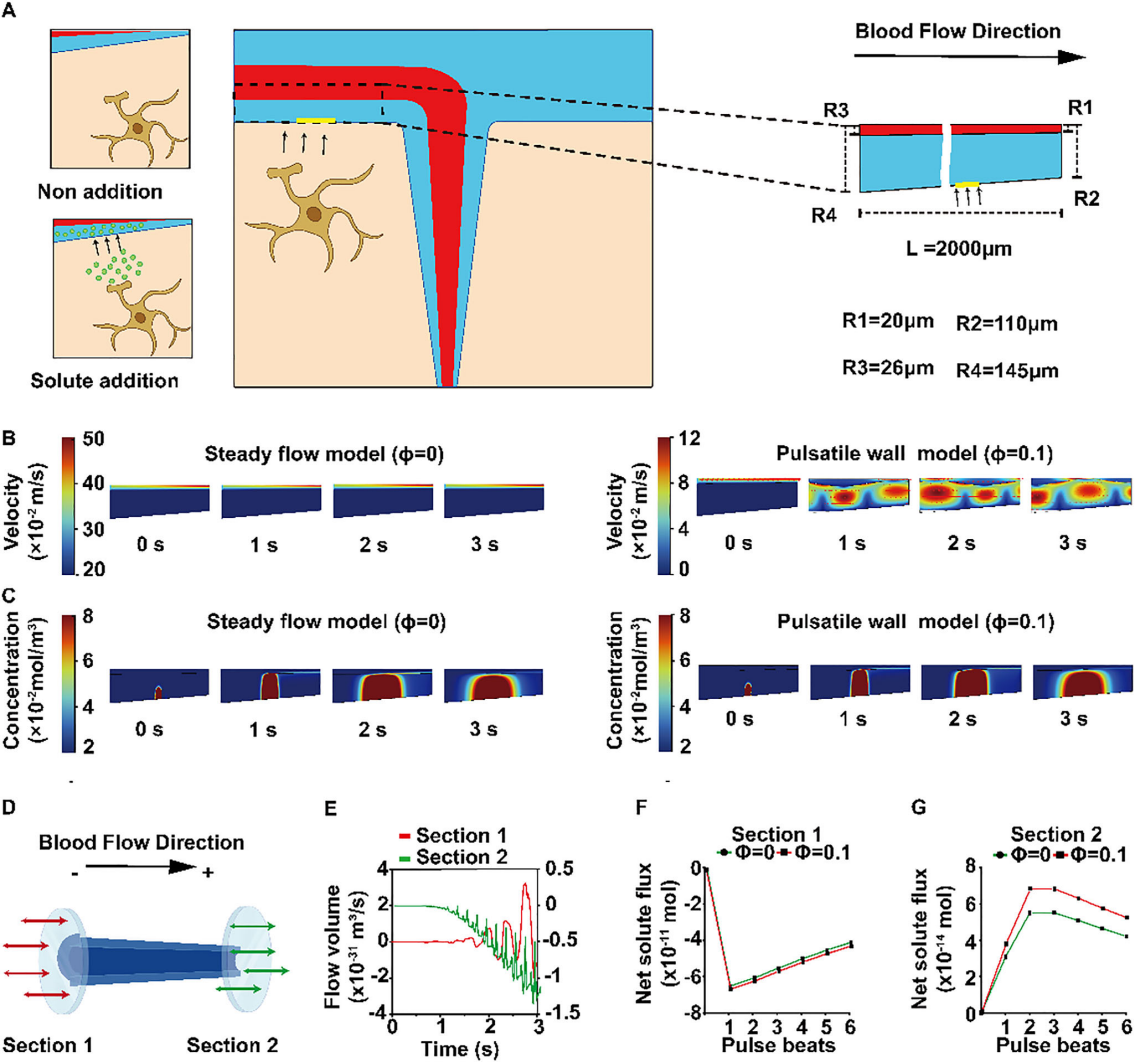
Simulation of solute molecule movement within the arterial PVS in the SAS. (A) PVS model constructed based on the physiological parameters of the middle cerebral artery. (B) Two‐dimensional simulation of cerebrospinal fluid (CSF) flow within the perivascular space (PVS) under non‐pulsatile (φ = 0) and arterial pulsatile (φ = 0.1) conditions. (C) Solute distribution following its injection at the midpoint of the PVS (left), and its transport over time under non‐pulsatile (φ = 0) and pulsatile (φ = 0.1) conditions (right). (D) Schematic defining Cross‐Sections 1 and 2 at the ends of the arterial PVS, with the sign convention for flow direction (positive downstream, negative upstream). (E) Instantaneous CSF flux across Section 1 (red) and Section 2 (green) over 0–3 s. (F) Cumulative net solute efflux across Section 1. (G) Cumulative net solute efflux across Section 2.

Simulations, based on MCA parameters [47], modeled pulsation‐driven CSF flow (Figure 5A). Based on the arterial PVS computational model established previously, we further introduced SAS boundary conditions to develop an extended model for characterizing fluid and solute exchange at the SAS‐arterial PVS interface (Figure 5A). The model retained the configuration of a proximal inlet and a distal outlet, under both non‐pulsatile (φ = 0) and arterial pulsatile (φ = 0.013) conditions. A comparison of the flow fields revealed distinct patterns (Figure 5B). Under the non‐pulsatile condition, a steady, laminar flow profile developed along the principal axis of the PVS. In contrast, the arterial pulsatile condition induced a prominent oscillatory flow field due to the periodic radial motion of the arterial wall, which exhibited quasi‐steady‐state characteristics during specific time windows.

Simulations of solute transport, following its injection at the PVS midpoint, showed that diffusion along the central axis was the primary, slow transport mechanism in the absence of pulsation. With pulsation, however, the solute underwent bidirectional oscillatory motion coupled with faster convective transport along the PVS axis, resulting in a wider distribution profile within the same timeframe (Figure 5C).

For analysis, the PVS ends were defined as Section 1 and Section 2, with flow direction relative to blood flow designated as positive (downstream) or negative (upstream) (Figure 5D). The simulated CSF flow within the PVS was oscillatory and synchronized with the cardiac cycle (Figure 5E). The velocity magnitude reached a quasi‐steady plateau around 1 s, stabilizing at approximately 2 units (outlet) and ‐0.5 units (inlet) under sustained pulsation.

Quantitative analysis further indicated a net solute efflux at both PVS ends under pulsatile conditions, but with significant asymmetry. The cumulative net solute flux was substantially higher at Section 1 than at Section 2 (Figure 5F,G), suggesting that solute exchange at the SAS‐arterial PVS interface was predominantly localized to Section 1 under the current model parameters and boundary conditions. This asymmetry likely stems from the modulation of the pulsation‐induced flow by the PVS geometry and boundaries, which alters the local coupling of convection and diffusion, leading to differential exchange efficiency between the two sections.

### 2.6 Pulsations Drive Aβ Clearance From the Arterial PVS

**FIGURE 6:**
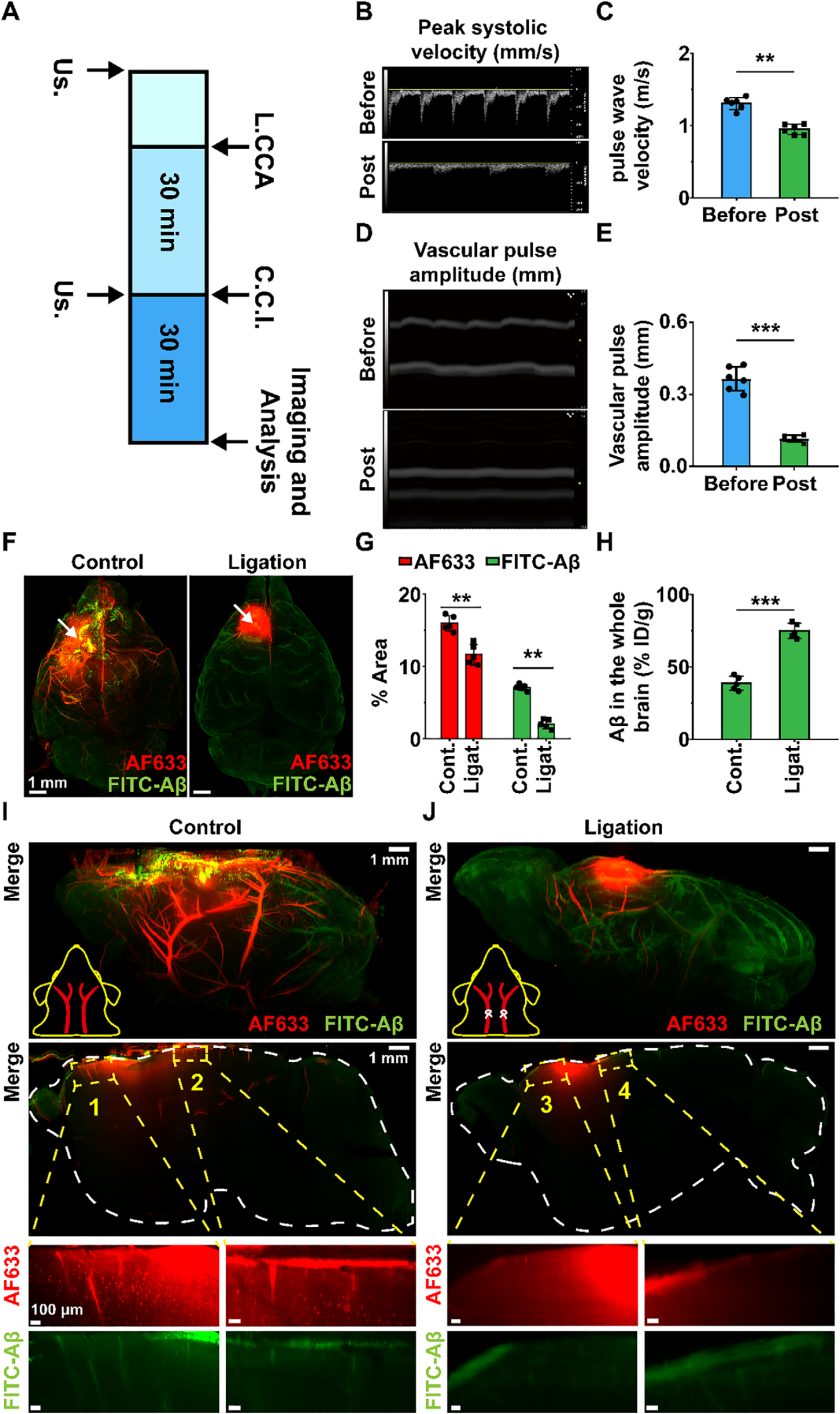
Arterial pulsation drives Aβ propagation in the brain parenchyma. (A) Schematic of experimental timeline: Cortical infusion followed by bilateral common carotid artery (BCA) ligation in mice, with assessments performed pre‐ and post‐ligation. (B) Representative peak systolic velocity ultrasound images and (C) pulse wave velocity (PWV) analysis pre‐ and post‐BCA ligation. (D) Vascular pulse amplitude traces and (E) corresponding quantitative analysis pre‐ and post‐ligation. (F) 3D‐rendered images showing FITC‐labeled Aβ (FITC‐Aβ) and reference tracer AF633 distribution. Quantitative analysis of (G) FITC‐Aβ distribution area and (H) parenchymal residual volume (mean ± SEM; n = 5 mice/group; *p* < 0.05, Student's t‐test). (I) Lateral 3D cleared brain images comparing FITC‐Aβ and AF633 distribution within the arterial PVS of control mice versus BCA‐ligated mice. Abbreviations: Cont, control group; L.CCA, ligation of common carotid artery; C.C.I., cerebral cortical infusion; Ligat., ligation group; Us., Ultrasound.

Arterial pulsations have long been considered the driving force for CSF movement [17, 47, 49]. To assess the impact of diminished arterial pulsation on amyloid‐beta (Aβ) distribution and retention in the brain, we performed bilateral common carotid artery ligation (Figure 6A). Ultrasound confirmed that ligation directly reduced the speed and amplitude of distal vessel pulsations (Figure 6B–E). Three‐dimensional cleared whole‐brain imaging revealed reduced FITC‐Aβ distribution areas (Figure 6F,G) and increased retention (Figure 6H) throughout the brain in ligated mice versus control mice. In control mice, AF633 signal was localized to the injection site and PVS (including the anterior cerebral artery), with abundant FITC‐Aβ signal also detected in arterial PVS (ROIs 1 and 2). Conversely, in ligated mice, AF633 and FITC‐Aβ signals were concentrated at the injection site, with negligible signal observed in arterial PVS (ROIs 3 and 4) (Figure 6I,J). These data indicate that arterial pulsation critically drives Aβ propagation from the infusion site along arterial PVS to distant brain tissues.

Consistent with our model of solute transport driven by arterial pulsation and basement membrane gradients, arterial ligation weakens both forces. This permits local parenchymal diffusion but impairs directed movement along arterial walls, leading to pronounced Aβ retention at the injection site and an overall increase in its residual burden in the brain.

## 3 Discussion

Our study indicates distinct solute transport mechanisms between the SAS and ventricular system compared to brain parenchyma. The CSF serves multiple roles, including the transport of metabolic waste, nutrients, and neurotransmitters, rather than merely acting as a drainage system for metabolic waste within the brain. Solute molecules in the SAS and ventricular system can both enter and exit the brain parenchyma. Within the parenchyma, solutes can also be transported toward the SAS, highlighting the dynamic interplay between these compartments and their roles in maintaining brain homeostasis.

In the cortical parenchyma, the PVS serves as a continuous anatomical conduit extending from arteries to veins, facilitating solute exchange between CSF, ISF, and the brain parenchyma. Our 3DISCO imaging following cortical tracer injection revealed tracer localization within both arterial and venous PVS compartments, indicating their dual involvement in parenchymal solute clearance. Structurally, as penetrating arterioles transition into capillaries, where the smooth muscle layer and pial sheath disappear, the arterial PVS becomes continuous with the capillary PVS, both of which directly adjoin the endothelial basal lamina, a thin extracellular matrix formed by endothelial cells and astrocytes. Further along the vascular tree, as capillaries converge into venules and veins, the fluid‐filled PVS expands, bounded internally by the venous wall and externally by the glial limitans. Functionally, within our proposed solute‐transport model, jointly driven by arterial pulsation and basement‐membrane concentration gradients, the PVS primarily enables macroscale transport and bidirectional mixing of solutes, propelling them toward the precapillary arterioles and into the more microscopic PVS. In turn, the PVS acts as a critical exchange interface, where basement‐membrane concentration gradients between precapillary arterioles and capillaries drive solute permeation along the basement membrane toward the venous end, ultimately enabling clearance. Additional evidence suggests that macromolecules may be transferred from arterial to venous PVS at sites where arteries and veins are in close apposition, thereby providing an alternative route for waste clearance [50].

Computational simulations show that CSF within the arterial PVS flows in the direction of blood flow under pulsatile conditions, facilitating solute transport from the SAS into the brain parenchyma. However, solute movement does not completely parallel the overall movement of CSF. Our simulation data demonstrate that solutes can undergo bidirectional transport within the arterial PVS during arterial pulsation, with transport quantities correlated with the magnitude of pulsation, highlighting the complex dynamics of solute transport influenced by arterial activity. This notion is consistent with the findings of Holstein‐Rønsbo et al., who demonstrated that neurovascular coupling directly enhances CSF flow via arterial dilation, thereby facilitating the clearance of metabolic waste [51].

Following CM injection, solute accessed the PVS within the SAS. Solutes routed through the Circle of Willis were transported along the ACA towards the olfactory mucosa, or along the MCA into the brain parenchyma. This demonstrates that arterial pulsation‐driven enhancement of net CSF flow along vessels is a key driver of solute transport from the subarachnoid PVS into the parenchyma. Cerebral cortical microinjection using glass microelectrodes [48, 49] provides a minimally invasive approach to study solute transport dynamics within the brain parenchyma. Employing this technique, we demonstrate bidirectional Aβ movement within the arterial PVS, enabling both anterograde and retrograde flow relative to blood flow direction. In control mice, Aβ injected into the parenchyma accesses the arterial PVS and is subsequently driven towards the SAS by arterial pulsation, resulting in efficient clearance from the injection site. In contrast, carotid‐ligated mice exhibit significantly elevated brain Aβ retention. This impaired clearance stems directly from reduced arterial pulsation amplitude, which diminishes both the diffusion of parenchymal Aβ into the arterial PVS and its subsequent pulsation‐driven transport towards the SAS.

## 4 Conclusions

Our study demonstrates that arterial PVS are the principal pathways for bidirectional solute exchange between the brain parenchyma and the SAS. This transport is primarily driven by arterial pulsation. However, the resulting net CSF flow shows marked regional heterogeneity: large‐caliber subarachnoid arteries generate strong net CSF flow in the direction of blood flow, providing the main driving force for solute entry from the SAS into the parenchyma, whereas net CSF flow along penetrating cortical arterioles is substantially weaker. Consequently, within the parenchyma, arterial pulsation acts mainly by enhancing solute diffusion along vascular basement membranes, thereby promoting solute transport from arterial PVS toward the capillary bed.

## 5 Materials and Methods

### 5.1 Materials

All animal studies were conducted under a protocol approved by the Institutional Animal Care and Use Committee. C57BL/6J female mice were kept in standard 12 h light/dark cycles and used for experiments at 10–12 weeks. After the completion of imaging experiments, all mice were euthanized with CO_2_.

### 5.2 Preparation of [^64^Cu]CuCl_2_

To assess the distribution of ^64^Cu^2+^ ions in vivo, we mixed [^64^Cu]CuCl_2_ (1 mCi in 0.1 M HCl) with 1 x artificial CSF containing 125 mM of NaCl, 25 mM of NaHCO_3_, 0.5 mM NaH_2_PO_4_, 2.3 mM CaCl_2_, 1.6 mM MgCl_2_, and 2.5 mM KCl to achieve a pH of 7.0‐7.2. We used 1–2 µCi of [^64^Cu]CuCl_2_ for infusion into the cortex (2 µL), intraventricular space (2 µL), and SAS, 2 µL for subsequent imaging studies.

### 5.3 Intraplantar Injection of [^64^Cu]CuCl_2_

Intraplantar injection was performed using aseptic technique under light anesthesia with isoflurane (4%–5% for induction, 2%–3% for maintenance). Briefly, the animal was placed on a soft pad, and the hind paw was cleaned with 70% ethanol. A 31‐gauge needle attached to a 1 mL syringe was inserted into the plantar surface of the hind paw, perpendicular to the skin. The needle was carefully advanced until it reached the plantar fascia, and then 10 µL of [^64^Cu]CuCl_2_ (20‐30 µCi) was injected. PET imaging and data analysis were further performed as described below.

### 5.4 The Preparation of Fluorescent Molecules Mixture

A mixture of two dye molecules was prepared for injections into different brain regions, followed by 3DISCO imaging. The composition was as follows: 10 mg mL^−1^ of DW 3000 (Dextran, Texas Red, 3000 MW, Sigma‐Aldrich) and 7 mg mL^−1^ of AF633. (Alexa Fluor 633, Thermo Scientific).

### 5.5 Surgical Procedures, Dye Molecule Infusion, and [^64^Cu]CuCl_2_ Infusion

The stereotaxic surgeries were performed on adult C57 mice under aseptic techniques and 1.5% isoflurane anesthesia with a flow rate of 350 mL/min. The bregma and lambda sutures of the skull were exposed by a longitudinal skin incision. According to a previous study [1, 2, 3, 4, 5, 6, 7, 8, 9, 10], a high‐speed micro‐drill was used to thin a circular area of skull for preparing a thinned‐skull cranial window (2‐3 mm in diameter) in live mice.

Dye infusion was performed using a small‐animal stereotactic frame (RWD Life Science Co.), which was connected to a microinjection adaptor for delivery of the dye solution via a Hamilton syringe. Tapered glass needles were prepared using a pipette puller (Sutter Instrument, Novato, CA, USA) through a single‐step heating protocol, resulting in tips with a diameter of approximately 30 to 50 µm. The glass tips were then carefully broken using forceps before being attached to the syringe using hot glue. Mineral oil was used to exclude any air in the syringe. The dye solution was loaded into the needle from the tip for injection. The glass needle was gently inserted into thinned‐skull cranial window. [^64^Cu]CuCl_2_ and dye solution was infused into different regions as follows: cerebral cortex perfusion site (needle at 30° to the skull, pointing towards the cerebellum): AP: 0.2 mm, ML: 1.0 mm, DV: 1.0 mm. Ventricular perfusion site: AP: 0.5 mm, ML: 1.35 mm, DV: 2.2 mm. The infusion was performed using a syringe pump for a total volume of 1 µL within 10 min. After being left in place for 5 min post‐infusion, the needle was retracted gently from the skull. The hole was sealed with dental cement, and the scalp was sutured.

### 5.6 Cisterna Magna (CM) Infusion of [^64^Cu]CuCl_2_ and Dye Mixture

CM infusion was performed using a stereotaxic apparatus (RWD Life Science Co.) under sterile conditions. Briefly, the animals were anesthetized with isoflurane (4%–5% for induction, 2%–3% for maintenance) and placed in a stereotaxic frame. Apply eye ointment (Puralube Vet Ointment, Dechra) and disinfect with povidone‐iodine (Dynarex) and 70% ethanol. For local anesthesia, administer bupivacaine 1 mg mL^−1^ and subcutaneous buprenorphine 0.05 mg kg^−1^ for the subcutaneous incision. Identify the occipital crest and incise the overlying skin (approximately 1 cm) to expose the cisterna magna. Dry the dura with a cotton swab. The glass needle was gently inserted, avoiding damage to the cerebellum and medulla. Secure the needle with dental cement and seal the tubing. The tracer infusion will then be performed.

### 5.7 Dynamic Positron Emission Tomography (PET) Imaging of [^64^Cu]CuCl_2_

Dynamic PET imaging was performed to monitor [^64^Cu]CuCl_2_ transportation after injection. The histogram files were divided into 28 frames: six 10 s frames, six 30 s frames, six 60 s frames, and ten 120 s frames. The ordered subset expectation maximization 3D/maximum and posteriori (OSEM3D/MAP) reconstruction algorithm was used for reconstructing dynamic PET images. In addition, to determine the time‐activity curves of different brain areas, region‐of‐interest (ROI) analysis of PET images was performed, and tracer uptake was calculated as the percentage of injected dose per gram of tissue (%ID g^−1^).

### 5.8 PET/CT Imaging of [^64^Cu]CuCl_2_

PET/CT imaging (Siemens Medical Solutions, Germany) was performed after dynamic PET scans for longitudinal monitoring of [^64^Cu]CuCl_2_ at various time points (e.g., 30 min, 3 h, 16 h, and 24 h) after infusion. Based on the calibration coefficient of the scanner, ROI data were generated from counts/pixel and the volume for determining time‐activity curves in different organs, which were then divided by the injected dose to calculate the final ROI uptake in %ID g^−1^.

### 5.9 Histology and Imaging

At 10 min after SAS infusion, intraventricular infusion, or cortex infusion, brains were removed, fixed for 12 h, and then immersed in 30% sucrose in PBS at 4°C until they sank. Brain sections (30 µm) were prepared using a freezing microtome (CM1950, Leica, Germany) and observed using the VS120 virtual microscopy slide‐scanning system (Olympus, Tokyo, Japan).

### 5.10 Live Brain Slice Preparation and Imaging

Brain tissues of healthy mice were dissected and sliced into 300 µm‐thick sections using a Vibratome (Leica VT1000S vibratome) filled with ice‐cold Krebs buffer. All brain slices were then incubated with DMEM medium containing fluorescent molecules for 30 min at 33°C in an oxygenated (95% O_2_ and 5% CO_2_) cutting solution, which contains the following components: NaCl (125 mM), KCl (2.5 mM), CaCl_2_ (2 mM), MgCl_2_ (1 mM), NaH_2_PO_4_ (1.25 mM), NaHCO_3_ (26 mM), D‐glucose (25 mM), sodium ascorbate (1.3 mM), and sodium pyruvate (3.0 mM), at a pH value of 7.2 and osmolality of 310–320 mOsm. Subsequently, imaging was performed using the VS120 virtual microscopy slide scanning system (Olympus, Tokyo, Japan).

The FDISCO procedure was carried out in accordance with the published protocol (2) with all steps conducted at 4°C, under gentle shaking. Prior to the clearing process, tetrahydrofuran (THF, Sigma‐Aldrich, USA) and dibenzyl ether (DBE, Sigma‐Aldrich, USA) were subjected to column absorption chromatography with basic activated aluminum oxide (Sinopharm Chemical Reagent Co., Ltd, China) to eliminate peroxides. Brain samples were sequentially dehydrated in THF solutions that were diluted in H_2_O to achieve graded concentrations: 50%, 70%, 80%, and 100 vol% (three times), with each step taking 12 h. All THF solutions were pH adjusted to 9.0‐9.5 with trimethylamine (Sinopharm Chemical Reagent Co., Ltd, Shanghai, China). Following dehydration, samples were immersed into pure DBE until the clearing stage. Brain imaging was performed using the light sheet fluorescence microscope (Ultramicroscope, LaVision BioTec, Germany). The software packages ImageJ and Imaris were utilized to analyze and generate 3D‐rendered images.

### 5.11 Simulation Model of Solute Transport in the PVS

We employed a two‐dimensional axisymmetric geometric model represented in cylindrical coordinates (r, z), with the rotational axis of symmetry at r = 0. The two‐dimensional model was rotated to form a three‐dimensional model. The blood vessel transitions from thick to thin, with radii of R1 = 12 µm and R2 = 1.5 µm for the rough and narrow boundaries, respectively. The radii of the PVS adjacent to the vessel are R3 = 20 µm and R4 = 2 µm. The total height of the computational domain is L = 100 µm (as per [reference]). To simulate the mass transfer process after solute addition to the CS), a solute inlet was placed at the outer wall at z = 50 µm.

During the simulation process, the finite element physics model mainly utilized the laminar flow (SPF) interface, the moving mesh (ALE) interface, and the dilute species transport (TDS) interface available in the COMSOL software. The laminar flow (SPF) model primarily calculated the flow of CSF in the PVS adjacent to the artery. The deforming geometry moving mesh (ALE) model primarily calculated the deformation of the vessel wall due to pulsation. The dilute species transport (TDS) model primarily simulated the mass transfer process of solutes in CSF, considering diffusion driven by concentration gradients and convective mass transfer due to CSF flow induced by arterial pulsation.

We employ a laminar flow model to investigate the flow of CSF. Assuming CSF is incompressible, we utilize the Navier‐Stokes equations (Equation 1) to describe the relationship between changes in fluid momentum, pressure acting on the fluid, and viscous forces. Here, μ represents fluid viscosity, P denotes pressure, and u signifies flow velocity. We assume CSF behaves as a Newtonian fluid, with viscosity remaining constant and changing only with shear rate.

$$\begin{array}{c} \rho \left(\frac{\partial \vec{u}}{\partial t}+\vec{u}\cdot \nabla \vec{u}\right)=-\nabla P+\nabla \left(\mu \left(\nabla \vec{u}+{\left(\nabla \vec{u}\right)}^{T}\right)\right) \end{array}$$

We employ the Arbitrary Lagrangian‐Eulerian (ALE) interface to describe the pulsatile deformation of the vessel wall with the heartbeat. The entire computational domain is defined as a deformable geometry. We specify the normal (perpendicular to the wall) grid displacement at the boundary of the vessel wall, with the displacement um calculated as follows: um = R11 − z * (R11 − R12) / L, where R1 is the radius of the vessel, a function of z, given by R1 = R11 − z * (R11 − R12) / L; φ represents the amplitude ratio of the vessel pulsation, where φR1 is the maximum amplitude of vessel pulsation; L denotes the height of the computational domain, set to L = 100 µm; z corresponds to the height coordinate in the cylindrical coordinate system (r, z); c is the propagation velocity of the pulsation, with c = 120 µm s^−1^ in this model. When L = 100 µm and c = 120 µm s^−1^, the pulsation period t = L/c = 0.833 s, indicating that pulsation occurs every 0.833 s, and the geometric shape of the computational domain returns to its original state, repeating the deformation process.

$$u_{m}=\phi R_{1}\sin\left[\frac{2\pi }{L}\left(z-ct\right)\right]$$

We employ the Transient Diffusion (TDS) model to compute solute transport. Solute transport consists of two components: (1) Diffusion flux J = −D▽c resulting from concentration gradients, where D represents the diffusion coefficient; (2) Fluid flow within the vessel during pulsation leads to solute convection (i.e., convective solute transport process u·▽c, where u is the velocity field of fluid flow). We express the change in solute concentration c using the following equation:

$$\begin{array}{c} \frac{\partial c}{\partial t}+\nabla \cdot J+u\cdot \nabla_{c}=0\\ J=-D\cdot \nabla_{c} \end{array}$$

For the laminar flow model, the upper and lower boundaries of the PVS representing the narrow and wide openings are set as open boundary conditions with zero pressure difference between them. Other boundaries are considered as walls, with the no‐slip condition applied. For the transient diffusion model, an inlet for solute injection is set at z = 50 µm on the outer wall of the computational domain. A solute with a concentration of 0.1 mol L^−1^ is injected during the initial 0.005 s. The upper and lower boundaries of the PVS representing the narrow and wide openings are set as outflow boundary conditions. Specific model parameters are provided in Table 1.

**TABLE 1:** Primary input parameters of the model.

Name	Expressions	Numerical value	Description
R1	1.5 (µm)	1.5 E‐6m	Arterial fine outlet radius
R2	2.0 (µm)	2.0 E‐6m	PVS fine outlet radius
R3	12 (µm)	1.2 E‐5m	Arterial thick outlet radius
R4	20 (µm)	2.0 E‐5m	PVS thick outlet radius
u_CSF	0.001 (Pa*s)	0.001 Pa·s	CSF viscosity
rho_CSF	1000 (kg m‐3)	1000 kg m^−3^	CSF density
c	120 (µm s^−1^)	1.2 E‐4 ms^−1^	Pulse speed rate
lamda	100 (µm)	1.0 E‐4 m	Blood vessel calculation domain length
D	1.4e‐6 (cm^2^ s^−1^)	1.4 E‐10m^2^ s^−1^	Diffusion coefficient
t_on2	Lam da/c	0.83333 s	Pulsation period
c0	0.1 (mol L^−1^)	100 mol m^−3^	
Q0_c0	0.1 (mol L^−1^)/0.005(s)	20000 mol (m^3^·s) ^−1^	

### 5.12 Statistical Analysis

The data is presented as mean ± standard deviation (S.D.). To determine differences among groups, one‐way analysis of variance (ANOVA) was employed. Student's t‐test was utilized to evaluate the significance of differences between each pair of groups. Statistical significance was considered at a *p*‐value of less than 0.05.

## Author Contributions

Shiyong Li, Ye Wang, Dawei Jiang, and Weibo Cai conceptualized the idea. Kaelyn V. Becker and Jonathan W. Engle produced and provided Cu‐64. Shiyong Li, Zhe Zhang, Yamei Yu, and Guangyu Jia performed tissue section and fluorescent imaging. Shiyong Li, Ye Wang, and Dawei Jiang performed PET imaging and all other studies. Shiyong Li, Ye Wang, Dawei Jiang, and Weibo Cai wrote the draft and all authors took part in the manuscript preparation.

## Funding

This work was supported, in part, by the National Natural Science Foundation of China (82471354, 82171335, 82060254, 22277031), University of Wisconsin‐Madison, the National Institutes of Health (P30CA014520), and Science and Technology Program of Jiangxi Province (20224ACB206017, 20204BCJL22050, 20213BCJ22012).

## Ethics Statement

All animal studies were conducted under a protocol approved by the Institutional Animal Care and Use Committee of the Second Affiliated Hospital of Nanchang University [Approval Number NCULAE‐2023061001]. All experiments were performed in accordance with the relevant guidelines and regulations of Nanchang University and the national law on animal care.

This study was approved by the Research Ethics Committee of Second Affiliated Hospital of Nanchang University [Approval No. IIT‐2023‐350].

## Conflicts of Interest

The authors declare no conflicts of interest. Weibo Cai is a member of the *Exploration* editorial board, and he was not involved in the handling or peer review process of this manuscript.

## Supporting information




**Supporting File 1**: exp270203‐sup‐0001‐SuppMat.docx.

**Supporting File 2**: exp270203‐sup‐0002‐SuppMovieS1.avi.

## Data Availability

All data is available in the main text or the supplementary materials. All data supporting the findings of this study is available from the authors upon reasonable request.
