# Supplementary material for: Arterial Perivascular Space‐Mediated Solute Transport in the Mouse Brain
Source: Exploration (Beijing). 2026 Jun 19:20250264. Online ahead of print. doi: 10.1002/EXP.20250264 (PMC13394722; doi:10.1002/EXP.20250264)
Supplement: Supplementary file 1 — Supporting File 1: exp270203‐sup‐0001‐SuppMat.docx. [file EXP2-9999-0-s002.docx]

Supplementary Materials

**This file includes:**

Supplementary Figure 1-6

Captions for Movies S1

**Other** **Supplementary Materials for this manuscript include the following:**

Movies S1


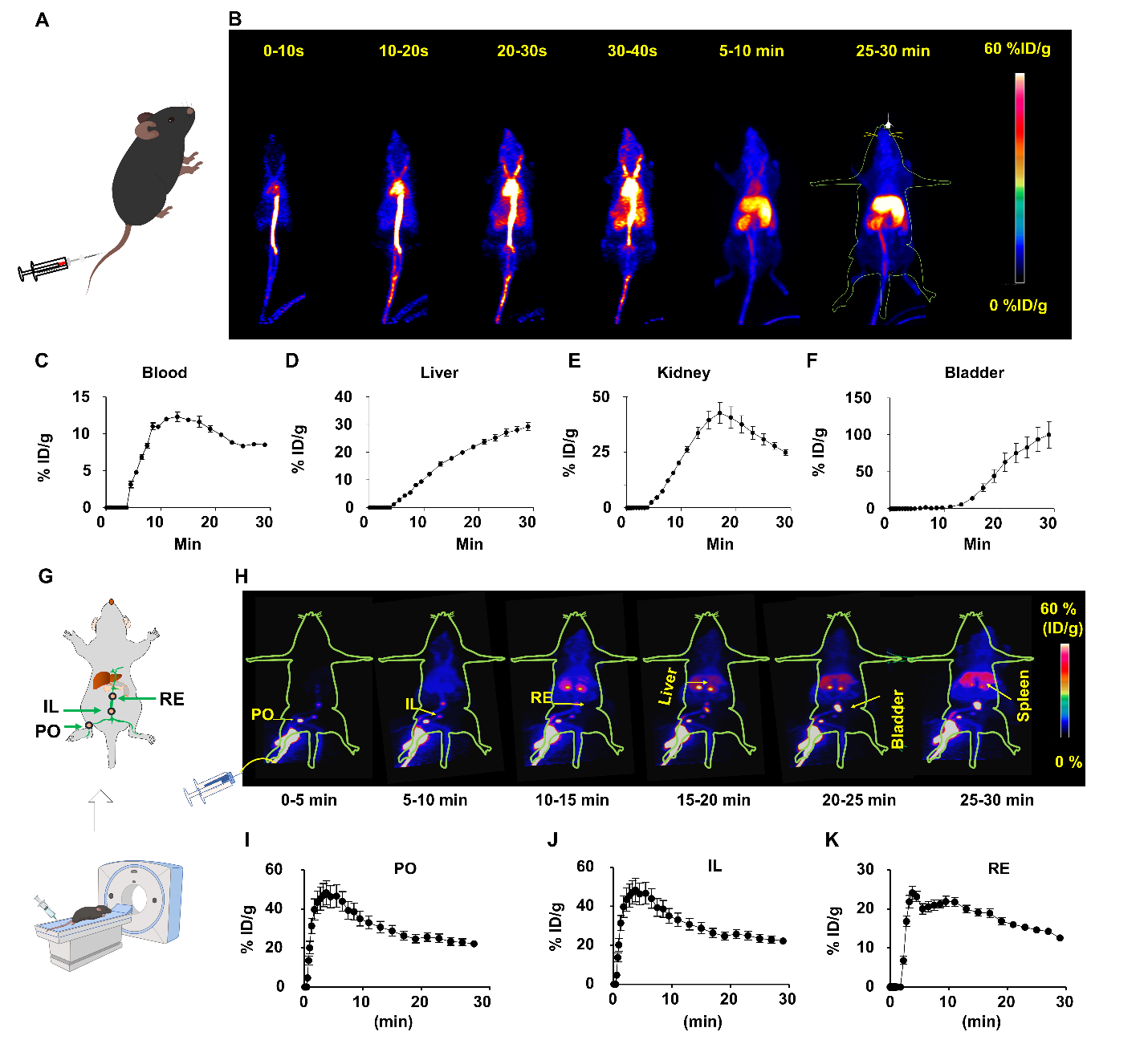


Supplementary Figure S1. Diagram (A), dynamic PET imaging (B) and time-activity curves of [^64^Cu]CuCl_2_ in different organs (C-F) after intraplantar injection. The delay of radioactivity uptake in all monitored organs suggested the slow absorption of [^64^Cu]CuCl_2_ in the lymphatic system and the fast clearance of [^64^Cu]CuCl_2_ from the blood circulation (n=3). (G) A schematic lymphatic drainage route of [^64^Cu]CuCl_2_ moving from PO (popliteal LN), IL (iliac LN) to RE (renal LN) after intraplantar injection. (H) Dynamic PET images at various time points after injection of [^64^Cu]CuCl_2_ into the mouse right hindpaw. Quantitative analyses of [64Cu]CuCl2 signal in the (I) PO, (J) IL, and (K) RE over 0-30 min after intraplantar infusion (n=3, mean ± SD, one-way ANOVA test).


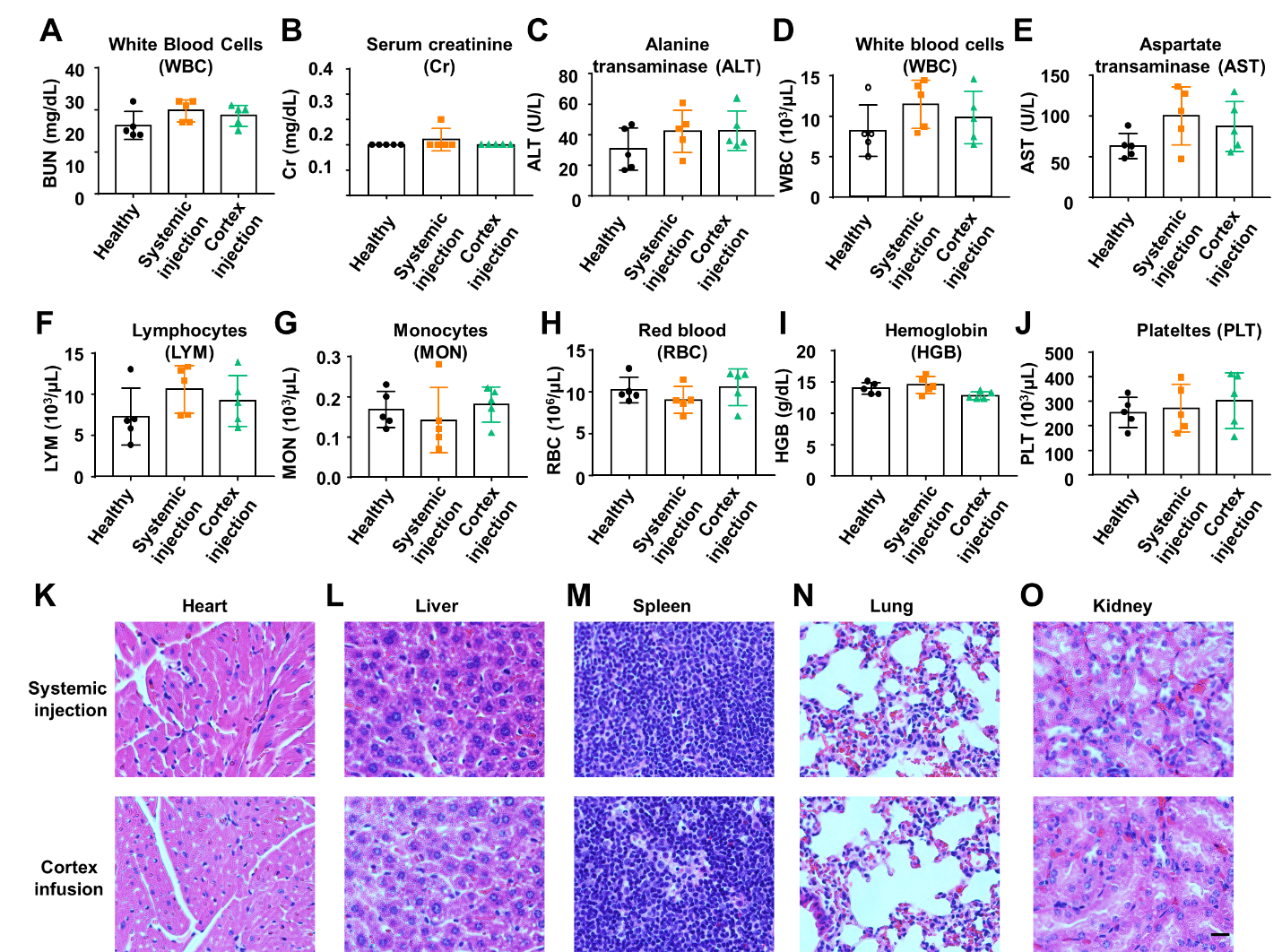


**Supplementary Figure S2. (A-J)** Serum tests of liver and kidney functions after systemic (tail vein) and direct cortex injection of [^64^Cu]CuCl_2_ at 24 h post-injection (n=5). Minimum functional changes were found after the injection, indicating lack of toxicity of [^64^Cu]CuCl_2_ infusion. (n=5, mean ± SD, one-way ANOVA test). **(K-O)** Hematological test results after systemic (tail vein) and direct cortex injection of [^64^Cu]CuCl_2_ at 24 h post-injection (n=5). Minimum changes were found after the injection, indicating lack of toxicity of [^64^Cu]CuCl_2_ infusion.


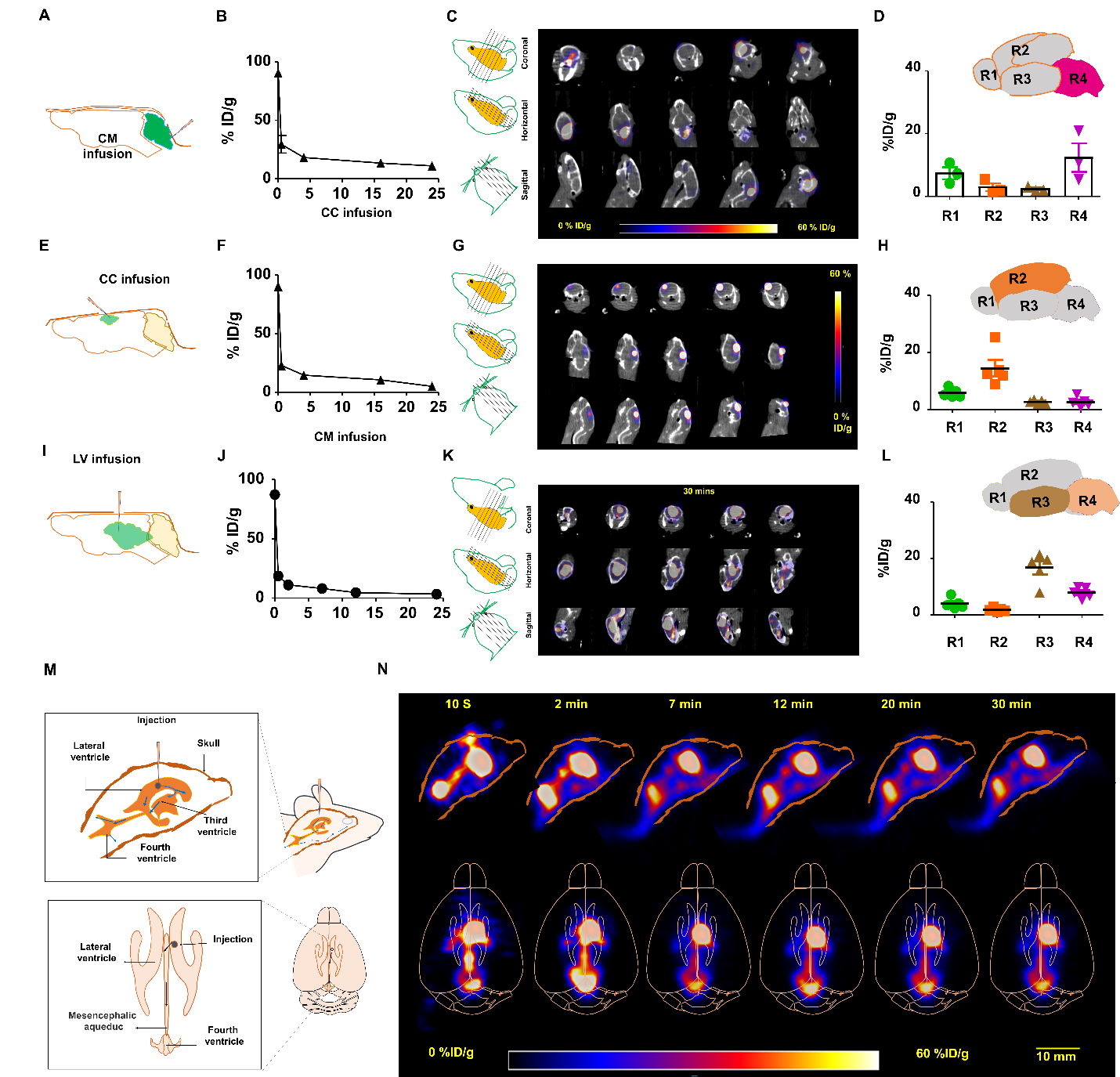


**Supplementary Figure S3. (A)** Schematic of CMI and **(B)** retention of [^64^Cu]CuCl_2_ in the brain. PET-CT images **(C)** and quantitative analysis **(D)** showing the distribution of [^64^Cu]CuCl_2_ in coronal, sagittal, and horizontal planes. **(E)** Schematic of CCI and **(F)** retention of [^64^Cu]CuCl_2_ in the brain. PET-CT images **(G)** and quantitative analysis **(H)** showing the distribution of [^64^Cu]CuCl_2_ in coronal, sagittal, and horizontal planes. **(I)** Schematic of LVI and **(J)** retention of [^64^Cu]CuCl_2_ in the brain. PET-CT images **(K)** and quantitative analysis **(L)** showing the distribution of [^64^Cu]CuCl_2_ in coronal, sagittal, and horizontal planes. (n=5, mean ± SD, one-way ANOVA test). (**M)** A schematic outline of the CSF outflow routes after intraventricular administration. (N) Representative PET images of [^64^Cu]CuCl_2_ at 10 s and 2, 7, 12, 20, and 30 min after intracortical injection.


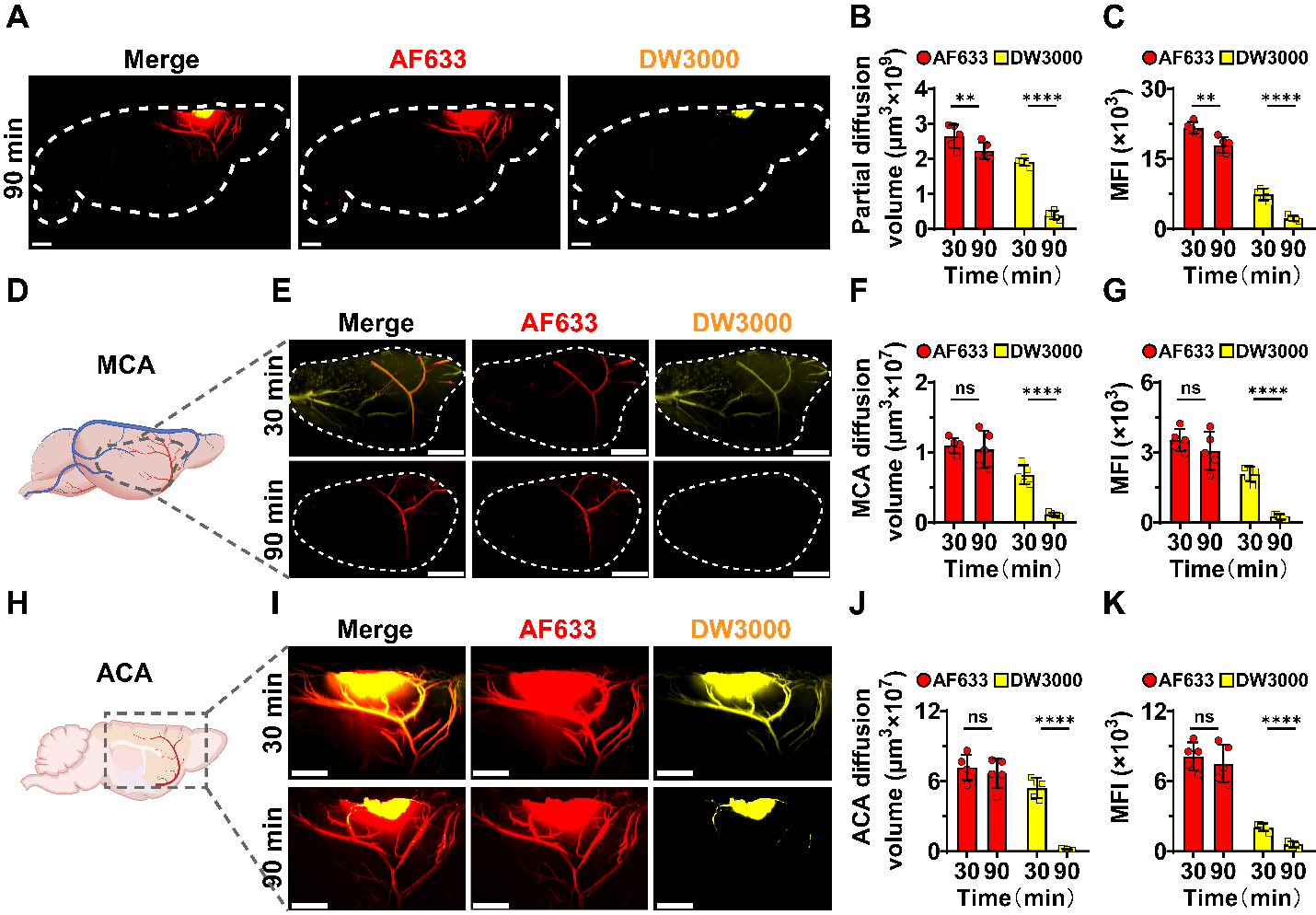


**Supplementary Figure S4.** Arterial perivascular spaces (PVS) mediate solute clearance from the cortical parenchyma.

(A) Schematic of 3DISCO imaging 90 min post-cortical injection of fluorescent tracers (AF633, red; DW3000, yellow). Whole brain (B) diffusion volume and (C) mean fluorescence intensity (MFI) of DW3000 and AF633. Solute transport pathways: schematic (D) and 3D imaging (E) (lateral view). Quantification of (F) diffusion volume and (G) residual tracer intensity in the middle cerebral artery (MCA). Solute transport pathways: schematic (H) and 3D imaging (I) (mid-sagittal view).(J, K) Quantification of (J) diffusion volume and (K) residual tracer intensity in the anterior cerebral artery (ACA).


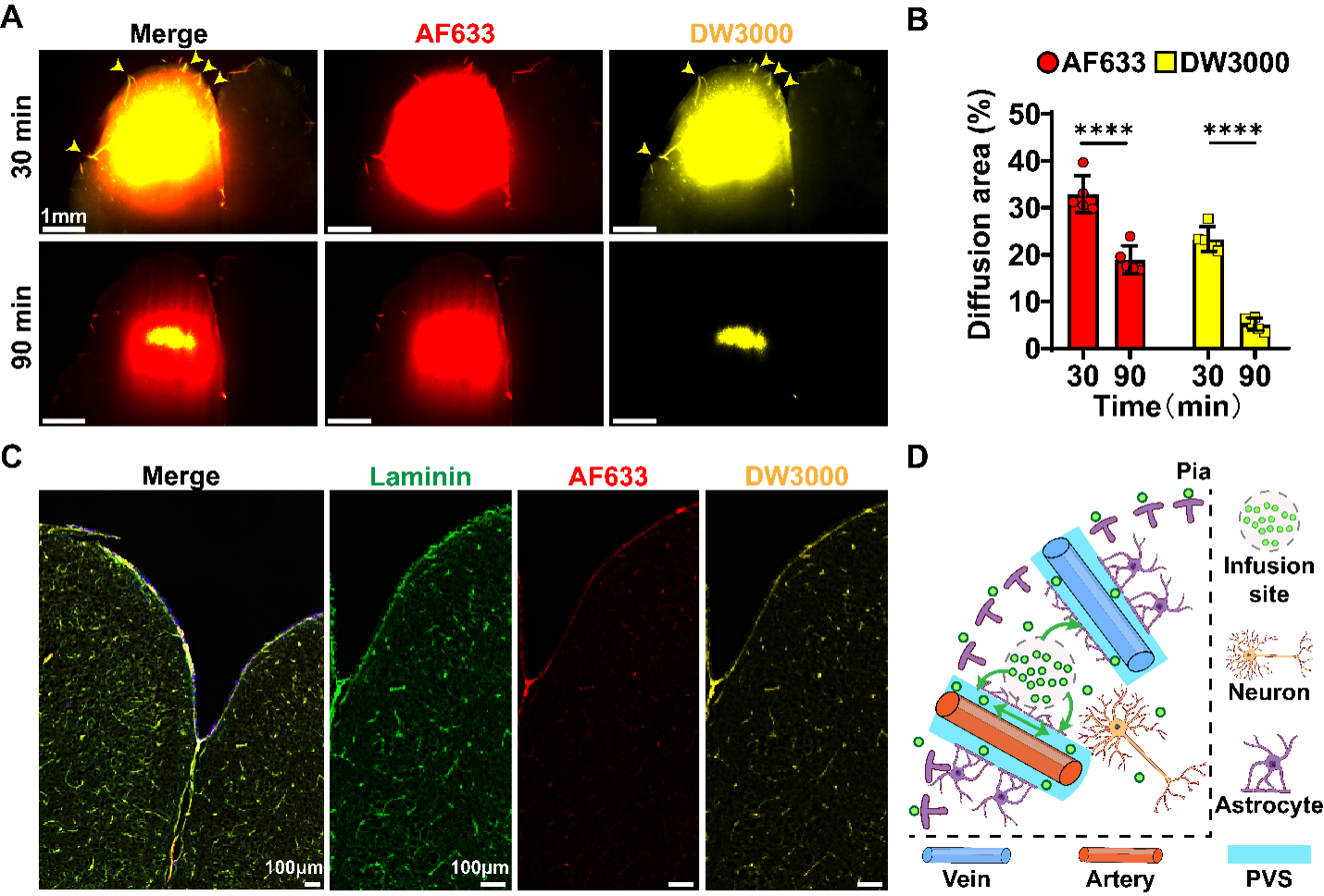


**Supplementary Figure S5.** Tracer distribution dynamics in perivascular spaces.

(A) Horizontal 3DISCO sections: DW3000 and AF633 distribution 30 min post-CCI (arrow: artery), Scale bar = 1 mm.. (B) Tracer distribution area (AF633, red; DW3000, yellow) at 30/90 min post-injection. (C) Perivascular colocalization of DW3000 (yellow) with the perivascular space of arteries (red) and the basement membrane (green) of capillaries and small vessels. Laminin (green) labels the basement membrane; AF633 (red) labels the arterial perivascular space. (D) Mechanistic schematic: solute exchange between parenchyma and perivascular spaces (arteries/veins).


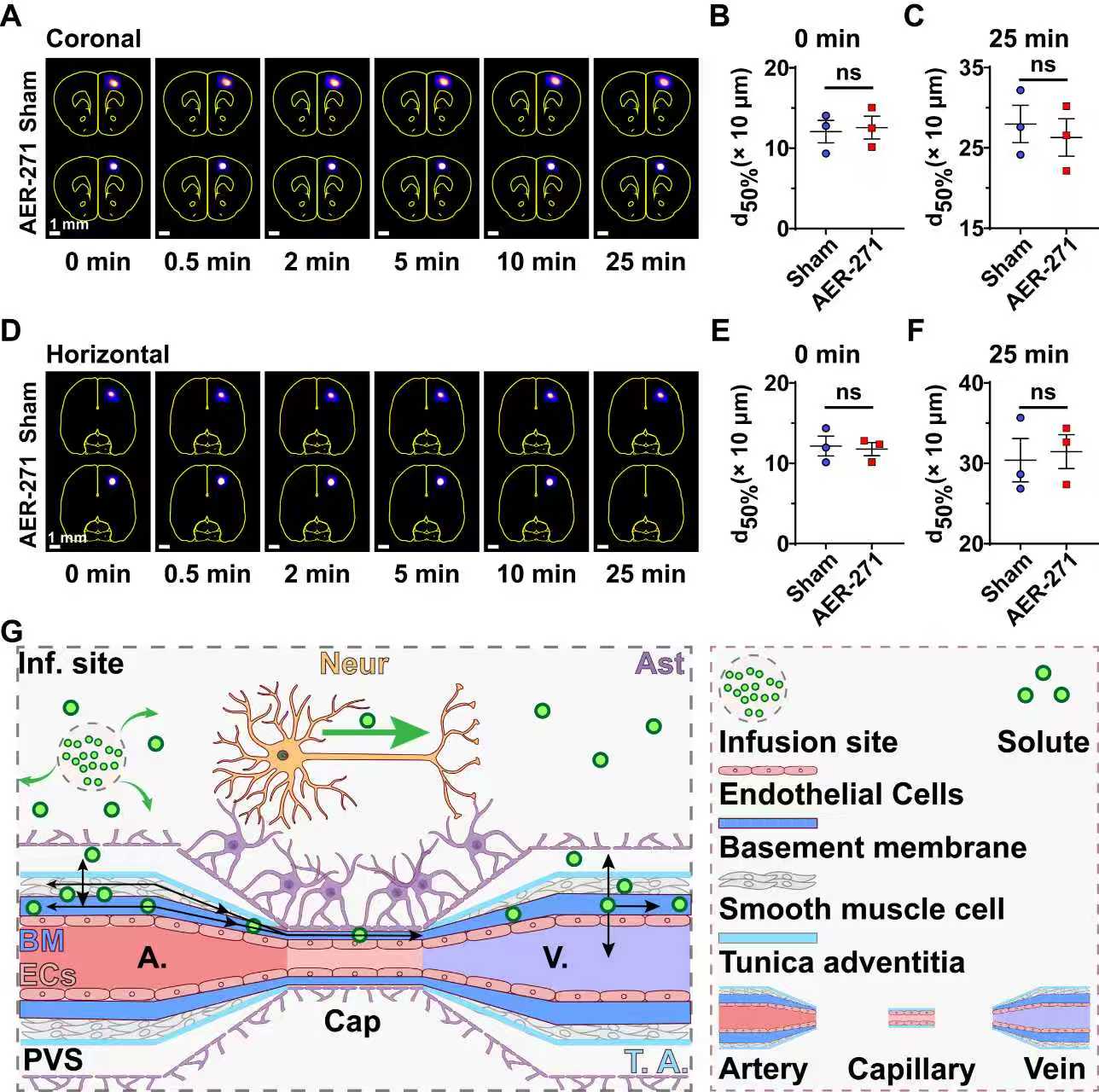


**Supplementary Figure 6** Dynamic PET imaging of [^64^Cu]CuCl2 distribution after cortical injection.

Representative coronal (A) and axial (B) brain PET/CT images acquired 0-25 min after cortical injection. (C-F) Quantification of the 50% diffusion distance of [^64^Cu]CuCl2 in the coronal (C, E) and sagittal (D, F) planes at 0 min (C, D) and 25 min (E, F) after injection in normal control mice and in mice pretreated with AOP4 inhibition. (G) Schematic of solute clearance pathways from the brain parenchymal extracellular space

Movie S1.

Dynamic PET imaging of ^64^CuCl_2_ in a healthy mouse after intraplantar injection.

**References**

[1] G. Yang, F. Pan, C. N. Parkhurst, J. Grutzendler, W. B. Gan, *Nat Protoc* **2010**, *5*, 201-208.
